# Genomic Sequence of a New *Alphavirus* Detected in Comber (Serranus cabrilla)

**DOI:** 10.1128/MRA.01294-19

**Published:** 2020-01-09

**Authors:** Gianpiero Zamperin, Adelaide Milani, Michele Gastaldelli, Rosita Quartesan, Andrea Fortin, Elisabetta Cappellozza, Pierpaolo Patarnello, Anna Toffan

**Affiliations:** a Istituto Zooprofilattico Sperimentale delle Venezie (IZSVe), Department of Comparative Biomedical Sciences, Legnaro, Padua, Italy; b Istituto Zooprofilattico Sperimentale delle Venezie (IZSVe), Specialistic Aquatic Animal Health Centre, Legnaro, Padua, Italy; c Istituto Zooprofilattico Sperimentale delle Venezie (IZSVe), Health Awareness and Communication Department, Legnaro, Padua, Italy; d Local Veterinary Services (ASL), Lecce, Italy; KU Leuven

## Abstract

The comber alphavirus was isolated from a fish cell line from the brain of an apparently healthy Serranus cabrilla specimen collected during wild fish surveillance in southern Italy. The comber alphavirus is a new member of the genus *Alphavirus*, family *Togaviridae*.

## ANNOUNCEMENT

**FIG 1:**
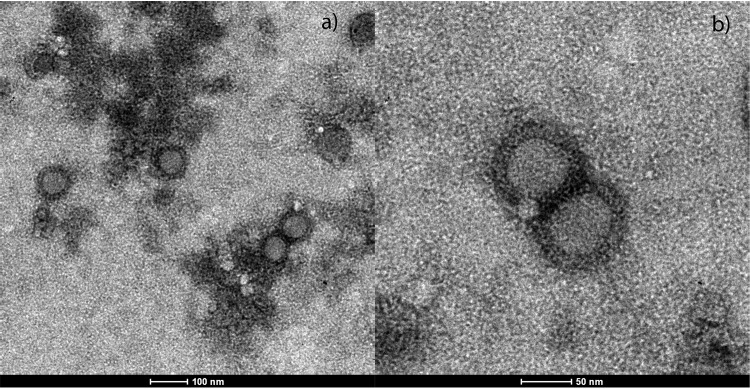
Togavirus-like particles observed with transmission electron microscopy (Philips EM208S) on BF-2 cell culture infected supernatant at magnifications of 22,000× (a) and 44,000× (b).

*Alphavirus* is the only genus presently included in the *Togaviridae* family. Alphaviruses are enveloped single-stranded positive-sense RNA genomes ranging from 9.7 to 11.8 kb (1). This genus comprises a large number of species that are mostly mosquito borne and pathogenic for mammals. In the aquatic environment, the species Salmonid alphavirus can cause severe diseases in Atlantic salmon and rainbow trout (1). Comber (Serranus cabrilla) is a small fish belonging to the family *Serranidae*, inhabiting the Mediterranean Sea. It can be found along the coast feeding on fish, cephalopods, and crustaceans. From 2010 to 2014, 32 *S. cabrilla* fish were caught during monitoring activity on the Salento coast (Ionian Sea). Comber brains were collected and subjected to viral isolation on a striped snakehead cell line (SSN-1) (2) at 25°C and a bluegill fry cell line (BF-2) (3) at 20°C according to standard procedures (4, 5). One sample produced clear cytopathic effect on both lines, with round and refractive cells detaching from the bottom of the flask up to complete monolayer destruction after 7 to 10 days. Transmission electron microscope observation revealed that the virus was pleomorphic, enveloped, and 55 to 70 nm in diameter with an icosahedral capsid 40 to 45 nm in diameter, resembling members of the *Togaviridae* family (Fig. 1). The new virus was called comber alphavirus isolate 12ITT-210.14.

Virions were precipitated from BF-2 cell supernatant with polyethylene glycol and then purified using ultracentrifugation on a 40 to 70% sucrose gradient to obtain a visible viral band, which was diluted in 10 ml of TRIS-HCl-EDTA-NaCl (TEN) buffer, pelleted by ultracentrifugation, and, finally, resuspended in 1 ml of TEN buffer (6). Total RNA was extracted using the total RNA isolation kit NucleoSpin RNA II (Macherey-Nagel) and converted into first- and second-strand cDNA using SuperScript III reverse transcriptase (Invitrogen) and second-strand cDNA synthesis (NEB), respectively. Sequencing libraries were generated using a Nextera DNA XT sample preparation kit (Illumina) and processed with a MiSeq reagent kit v2 (2 × 250-bp paired-end [PE] mode; Illumina). Sequencing yielded 1,138,673 251-bp-long paired-end reads, which were quality filtered by (i) removing reads with more than 100 bases with a Q score below 7 and duplicated paired-end reads, (ii) clipping adaptors with Scythe v0.991 (https://github.com/vsbuffalo/scythe), (iii) trimming low-quality 3′ ends with Sickle v1.33 (https://github.com/najoshi/sickle), and (iv) discarding reads shorter than 80 bases and unpaired reads. After filtering, we recovered 911,779 high-quality reads, which were taxonomically classified by (i) alignment against the integrated nucleotide (NT) database (26 January 2016 version) using BLAST v2.3.0+ (7) with default parameters and against the integrated nonredundant (NR) database (26 January 2016 version) using DIAMOND v0.8.16 (8) with default parameters, (ii) filtering out alignment hits with an E value larger than 1 × 10^–3^, and (iii) feeding the remaining alignment hits to MEGAN v6.5.8 (9). Reads belonging to the *Alphavirus* genus were selected and *de novo* assembled using IDBA-UD v1.1.1 (10) with default parameters and the multi-*k*-mer approach (minimum value, 114; maximum value, 124; increment, 10). Only a single contig with a length comparable to the size of the *Alphavirus* genome was obtained. All reads belonging to the *Alphavirus* genus were subsequently aligned against the longest contig obtained from the *de novo* assembly using Burrows-Wheeler Aligner (BWA) v0.7.12 (11) with standard parameters. The alignment was manually revised with Tablet (12) to avoid the risk of misassembly.

The final genome obtained resulted in a consensus sequence 11,604 nucleotides long showing a sequence coverage of 30,221-fold and a 56.4% G+C content. A comparison performed using BLAST (online version, blastn algorithm) with full *Alphavirus* genomes available in GenBank (July 2019) revealed 59% query coverage and 67.2% identity with *Salmonid alphavirus* subtype 3 (GenBank accession number KC122924).

Open reading frames (ORFs) were determined using the NCBI tool ORFfinder (https://www.ncbi.nlm.nih.gov/orffinder/). It detected two distinct ORFs, an ORF in the 5′ two-thirds of the genome and a second one in the 3′ one-third of the genome, in agreement with the genomic content organization of other known alphaviruses.

Herein, we report the first description and the almost full-genome sequence of a new member of the genus *Alphavirus*, isolated from a wild Mediterranean fish.

### Data availability.

MiSeq raw data were submitted to the NCBI Sequence Read Archive (SRA) under the accession number SRR9718464. The comber *Alphavirus* genome sequence has been deposited in GenBank under the accession number MN207265.
